# dendsort: modular leaf ordering methods for dendrogram representations in R

**DOI:** 10.12688/f1000research.4784.1

**Published:** 2014-07-30

**Authors:** Ryo Sakai, Raf Winand, Toni Verbeiren, Andrew Vande Moere, Jan Aerts

**Affiliations:** 1 Department of Electrical Engineering (ESAT) STADIUS Center for Dynamical Systems, Signal Processing and Data Analytics, KU Leuven, 3001, Belgium; 2 iMinds Medical IT, KU Leuven, 3001, Belgium; 3 Department of Architecture, Research[x]Design, KU Leuven, 3001, Belgium

## Abstract

Dendrograms are graphical representations of binary tree structures resulting from agglomerative hierarchical clustering. In Life Science, a cluster heat map is a widely accepted visualization technique that utilizes the leaf order of a dendrogram to reorder the rows and columns of the data table. The derived linear order is more meaningful than a random order, because it groups similar items together. However, two consecutive items can be quite dissimilar despite proximity in the order. In addition, there are 2 ^n-1^ possible orderings given n input elements as the orientation of clusters at each merge can be flipped without affecting the hierarchical structure. We present two modular leaf ordering methods to encode both the monotonic order in which clusters are merged and the nested cluster relationships more faithfully in the resulting dendrogram structure. We compare dendrogram and cluster heat map visualizations created using our heuristics to the default heuristic in R and seriation-based leaf ordering methods. We find that our methods lead to a dendrogram structure with global patterns that are easier to interpret, more legible given a limited display space, and more insightful for some cases. The implementation of methods is available as an R package, named ”dendsort”, from the CRAN package repository. Further examples, documentations, and the source code are available at [https://bitbucket.org/biovizleuven/dendsort/].

## Introduction

Agglomerative hierarchical clustering (HC) is one of the classic and yet still very popular cluster analysis methods in data exploration ^1, 2^. Its implementation is widely available and execution of the clustering requires only a few settings, such as a choice of distance metric and linkage algorithm ^3^. The clustering process begins with individual input elements as singleton clusters and successively merges a pair of most similar clusters until only one cluster remains. The dissimilarity, or the distance, between two clusters is defined by a distance metric and updated by a linkage algorithm. The output of HC is typically represented in a form of a binary tree, called a dendrogram. In a dendrogram, the similarity of two clusters is encoded in the height of the branch where two clusters merge. Two very similar elements are merged in the early stages of clustering, thus the height of the branches between these elements is relatively small. The dissimilarity between two clusters increases with each successive merge, resulting in a binary hierarchical structure with a monotonic property ^4^. Therefore, a dendrogram represents both cluster-subcluster relationships as well as the order in which the clusters were merged ^5^.

**Figure 1.**
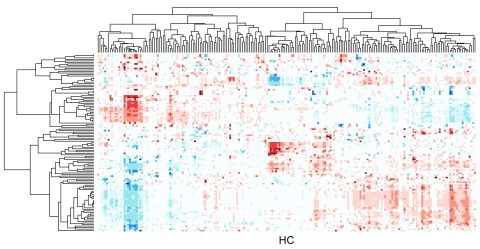
Cluster heat map of the data matrix from the integrated pathway analysis of gastric cancer from the Cancer Genome Atlas (TCGA) study.

There are two unique uses of a dendrogram in exploratory data analysis. First, clusters of input elements can be inferred from the subtree structures below a certain threshold by “cutting the tree”. It is an advantage of hierarchical clustering that this threshold value can be adjusted based on domain-specific knowledge to result in clusters of different sizes. Second, a linear order of observations (rows) or attributes (columns) of an associated matrix can be derived. This linear order of observations is typically used to reorder the columns or rows of the data matrix. Then, the matrix is visualized as cluster heat maps ^1^, where dendrograms and heat map visualizations are coupled ( Figure 1).

The linear order derived from a dendrogram is more meaningful than a random order, as it groups similar items together ^6, 7^. However, two consecutive items in this order are not necessarily similar, since these leaves could belong to different subtree structures, or simply be quite distant from each other. This is a common misinterpretation of a dendrogram: one may expect similarity between two input elements based on the proximity in the leaf order ^8, 9^. In addition, there are 2 *n−*1 possible orderings given *n* input elements, because the orientation of clusters at each merge can be flipped without affecting the underlying hierarchical structure, thus rendering a unique optimization challenge.

To address the misinterpretation of dendrograms and the optimization problem, a number of methods have been proposed to rearrange the structure of a dendrogram. Gruvaeus and Wainer ^10^ proposed a method (GW) to order leaves such that two singleton clusters at the edge of adjacent subtrees are most similar, given the constraint of the binary tree structure. Bar-Joseph *et al.* ^6^ proposed a method, called the optimal leaf ordering (OLO), to maximize the sum of the similarity of any adjacent elements in the ordering. Similarly, Chae and Chen ^11^ proposed a method for ordering by minimizing the bilateral symmetric distance between two adjacent clusters. All these methods aim to homogenize the linear order in one way or another and are evaluated in terms of either a loss function, such as the Hamiltonian path length, or a merit function, such as the number of anti-Robinson events ^12^.

Even though these seriation-based leaf ordering methods exploit the binary tree structure to reduce the number of permissible permutations, these methods have short-comings. First, they homogenize and optimize the distance between items in the linear order, and this still encourages the common misinterpretation of dendrograms, reading a dendrogram horizontally. Second, the dendrogram structure is only a means to reduce the number of permissible permutations, and the graphical representation of the resulting dendrogram obscures the intrinsic properties of the hierarchical clustering result, such as the cluster-subcluster relationship and the order in which clusters are merged.

In the biological domain, Eisen *et al.* ^13^ have introduced and established a cluster analysis method for high throughput gene expression data using cluster heat maps. The method includes a leaf orderings by weighting genes based on genome coordinates or the average expression level. The resulting linear order is more meaningful in terms of biology, but the method requires prior knowledge or additional information for the weighting.

In this paper, we present leaf ordering heuristics, named modular leaf ordering (MOLO), to address the aforementioned shortcomings by constructing a dendrogram that reflects a) the monotonic order in which clusters are merged and b) the nested cluster relationships. We compare dendrogram and cluster heat map visualizations created using our heuristics to the default heuristic in R and seriation-based leaf ordering methods. The implementation is available as an R package, named "dendsort", from the CRAN package repository. The R script for generating figures in this paper is available as a supplementary material. Further examples, documentations, and the source code are available at [ https://bitbucket.org/biovizleuven/dendsort/].

## Methods

### Hierarchical clustering

Agglomerative hierarchical clustering (HC) starts with individual observations as singleton clusters and merges clusters iteratively until all clusters belong to one big cluster. In each iteration, the two most similar clusters are identified by a distance measure and a linkage algorithm of choice. The details of the algorithm and the properties of distance measures and linkage algorithms are described in ^4, 5, 14^.

The default hierarchical clustering method in R combines three types of merges: a merge between two singleton clusters, a merge between a singleton cluster and a cluster with more than one member, and a merge between clusters with multiple members. The heuristics for determining the orientation of merging elements essentially determine the structure of the resulting dendrogram.

**Figure 2.**
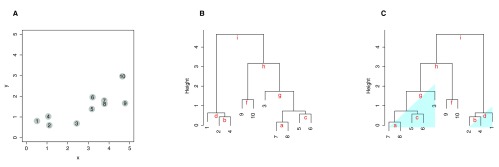
Hierarchical clustering of a simulated two-dimensional data set. ( **A**) A scatterplot of the ten input elements. The number of each element also represents the order in the input matrix. ( **B**) A dendrogram drawn using the default heuristics in R. The branches in the dendrogram are labeled from “a” to “i” in the order in which clusters are merged. ( **C**) A dendrogram reordered using MOLO with the smallest distance. The global structures in a shape of the right triangle are highlighted.

Using a simple two-dimensional data set as shown in Figure 2A, we demonstrate the default heuristics used in the hierarchical clustering method in R. A dendrogram is constructed as follows: When a leaf (singleton cluster) merges with another leaf, the orientation of clusters is determined by the order of observations in the input data matrix, as seen in branch “a”, “b”, “c” and “f” in Figure 2B. When a leaf merges with a cluster with more than one member (subtree), the leaf is always placed on the left side of the branch, as shown in branch "d" and "g". When two subtree merges, the subtree with the smaller distance in the previous merge is placed on the left, as seen in branch “e”, “h”, and “i”. Each branch is labeled alphabetically in the order of merges within the clustering process.

In contrast to the default heuristics, our heuristics are characterized by 2 key differences: first, a leaf is placed on the right side when it merges with a subtree; second, when two subtrees merge, the subtree with the smallest distance among all of preceding merges is placed on the left ( Figure 2C). The first rule avoids a branch of a singleton cluster hanging over the preceding nested clusters and allows the tree to grow from left to right in the order of merges. The second rule ensures that the tightest cluster is placed leftmost within the subtree structure. Consequently, our heuristics result in each subtree or sub-cluster structure in a right triangular shape, as shown in Figure 2C. This feature increases the contrast between the items at the edge of adjacent subtree structures, thus modularizing each subtree structure.

**Figure 3.**
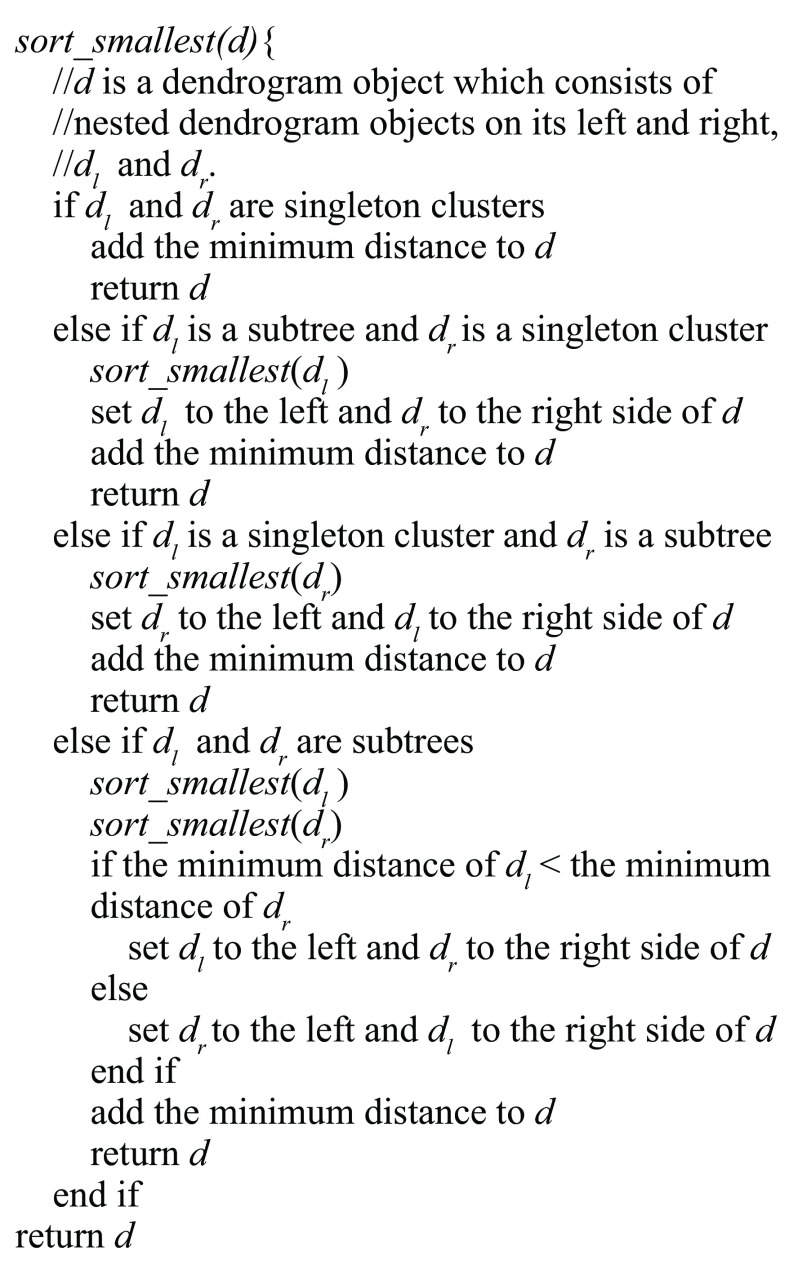
The recursive algorithm for ordering a dendrogram structure based on the minimum distance.

The MOLO method takes the result of the default hierarchical clustering method, and reevaluates the orientation of the clusters at each branch recursively. The pseudocode of this algorithm is shown in Figure 3. In addition to the algorithm based on the smallest distance, we also implemented a variant in which the average distances of all preceding merges are compared, and discussed further in the third case study. The data in Figure 2 consist of only 10 observations and it is merely intended to explain the difference in heuristics. Following case studies demonstrate applications of the MOLO algorithm with larger datasets, and compare visualizations created using our heuristics and other existing leaf ordering methods.

## Results

### Case study 1: Comparison of clustering algorithms

One of the key tasks in applying hierarchical clustering is to choose an appropriate distance metric and a linkage algorithm ^14^. A choice of distance metric, such as Euclidean distance and correlation-based distance, defines a measure of similarity between two elements. Clustering algorithms, such as complete, average, and single linkage, are variations of the cluster proximity definition ^5^. The choice of distance measures and linkage algorithms influences the clustering results. It is therefore recommended to try different HC settings in exploratory data analysis, especially when the underlying data structure is unknown.

**Figure 4.**
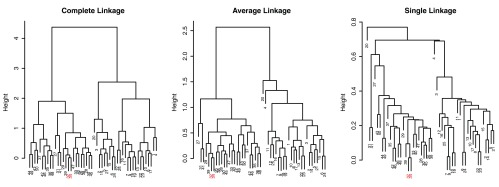
Comparison of dendrograms from different linkage algorithms using R’s default ordering heuristics. The element 32 and 34 are highlighted.

**Figure 5.**
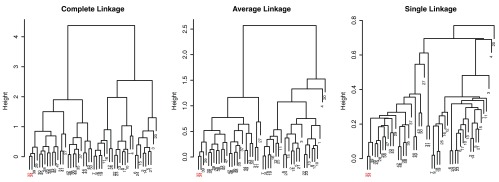
Comparison of dendrograms from different linkage algorithms after applying the MOLO method based on the smallest distance. The element 32 and 34 are highlighted.

As Hastie *et al.* ^4^ point out, dendrogram structures can vary greatly depending on the choice of linkage algorithms. In Figure 4, dendrograms of different linkage algorithms for the same simulated data set are compared. The appearance of the dendrogram structure is quite different and it is difficult to compare similarities in the nested cluster structure. In contrast, when the MOLO method is applied, we find the reordered dendrograms easier to study the nested structure and to compare between one another ( Figure 5), because the linear leaf order in these dendrograms reflect the order in which clusters are merged. For instance, the element 32 and 34 form the tightest cluster, and they are easy to identify because they are always placed leftmost. Also, upon closer examination of the reordered dendrogram structures, we find that the reordered dendrograms reflect the underlying difference in algorithms more closely. For example, the average linkage is an intermediate approach between the single and complete linkage algorithms to define cluster proximity ^5^. Although the MOLO method does not change the clustering result itself, this case study demonstrates how it can improve, or at least bring a new perspective, to interpret dendrogram structures.

### Case study 2: Iris data

**Figure 6.**
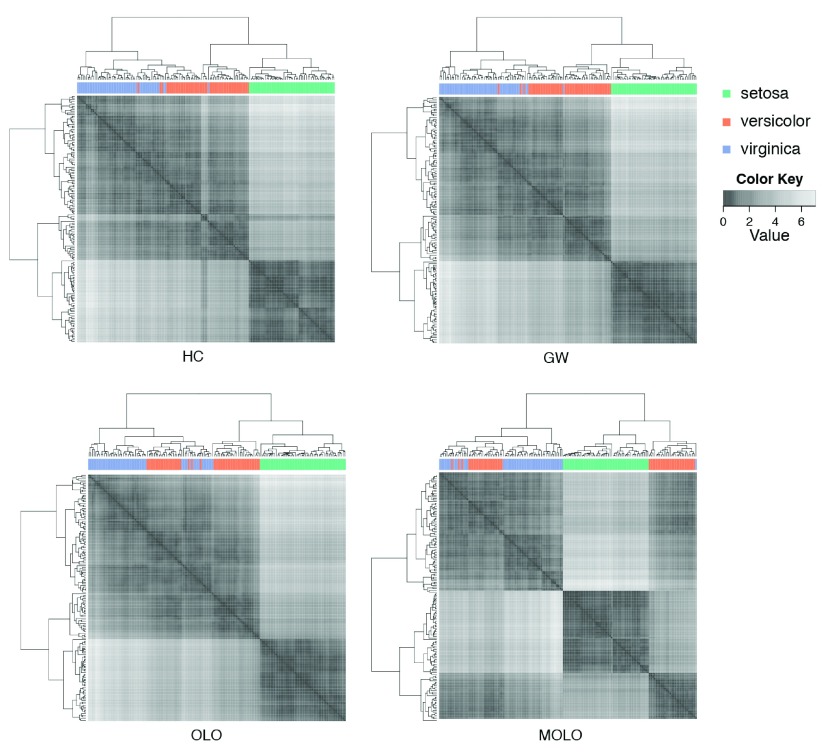
Comparison of leaf ordering methods in cluster heat maps. The default hierarchical clustering (HC), the Gruvaeus and Wainer’s method (GW), the optimal leaf ordering (OLO), and the MOLO method are applied to the Fisher’s Iris data set.

The second case study extends the demonstration of seriation-based leaf ordering methods by Buchta *et al.* ^15^ using the Fisher’s Iris data set. The Fisher’s Iris data set is available from the R’s *dataset* package ^16^. This Iris data set represents 3 species of iris with 50 observations for each species. Each observation contains measurements of 4 attributes: the sepal length and width, and the petal length and width. In this case we performed hierarchical clustering on the distance matrix of Euclidean distances, using the complete linkage algorithm. In Figure 6, adjacency matrices are visualized as cluster heat maps to compare results of the default hierarchical clustering (HC), the Gruvaeus and Wainer’s method (GW) ^10^, the optimal leaf ordering (OLO) ^6^, and the MOLO method (MOLO). These matrices are diagonally symmetric and rows and columns are reordered based on the leaf order of dendrograms. The species for each observation is color coded and shown between the dendrogram and the heat map visualization. Implementations of the GW and OLO methods are available in the *seriation* R package ^15^.

Despite the fact that each representation shares the same underlying hierarchical clustering output, the visual impressions of heat maps are different depending on the choice of leaf ordering methods. For example, the results of the HC, GW, and OLO methods suggest two predominant clusters, as indicated by dark square blocks along the diagonal axis. On the other hand, the result of the MOLO method suggests three clusters. The MOLO heuristics place the most similar items on the left ends of each subtree structure and subsequently merged clusters are placed on its right. As a result, the MOLO method reorders the dendrogram structure to reflect the modularity of the cluster-subcluster structure. With the information of species for each observation, it becomes clear that there are three species and a half of *versicolor* samples are clustered together with *virginica*.

Additionally, we find the cluster edges in the heat map visualization of the MOLO method are more prominent than those of other leaf ordering methods. One explanation for the enhanced edges is the increased contrast between subtree structures, whereas the GW and OLO methods aim to reduce the edge contrast between sub-tree structures, resulting in more fuzzy boundaries. This effect can be seen at the borders between *versicolor* and *virginica* species in heat map visualizations. The second explanation is that the monotonic linear order results in an optical illusion, called Mach band effect, at the edge of subtree structures. The Mach band effect explains how edges in different shades of gray have exaggerated contrast when in contact ^17^. This enhanced edge-detection works to our advantage in identifying clusters, especially because our visual systems to decode quantitative or continuous data from different shades of colors is limited ^18^.

As also pointed out in previous studies ^6^, the GW and OLO methods result in a global structure where highly similar items appear in the middle, while marginally related items are on the edge of the subtree structure. This tendency is most apparent in the *setosa* samples. On the other hand, the MOLO method results in a right triangular global shape where the similarity of clusters increases from left to right, unidirectionally, for each subtree structure. This global property enhances the contrast at the borders of clusters and reveals the third cluster in the heatmap visualizations.

### Case study 3: TCGA

The third case study involves a multivariate table obtained from the integrated pathway analysis of gastric cancer from the Cancer Genome Atlas (TCGA) study ^19^. In this data set, each column represents a pathway consisting of a set of genes and each row represents a cohort of samples based on specific clinical or genetic features. For each pair of a pathway and a feature, a continuous value of between 1 and -1 is assigned to score positive or negative association, respectively. The goal of this cluster analysis is to explore patterns in the data set and examine clusters to characterize the link between the gene expression levels and clinical features and to identify subtypes of the cancer among the cohort of samples.

These matrices are typically visualized as cluster heat maps ( Figure 1). By applying hierarchical clustering on the rows and columns independently, the rows and columns are reordered to place similar items close to each other. In this example, the distance measure is based on the Pearson’s Correlation coefficient and the complete linkage algorithm is used for hierarchical clustering.

**Figure 7.**
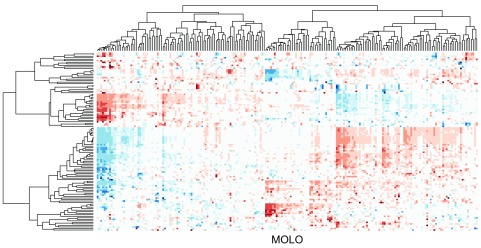
Cluster heat map of the data matrix after applying the MOLO method based on the smallest distance.

**Figure 8.**
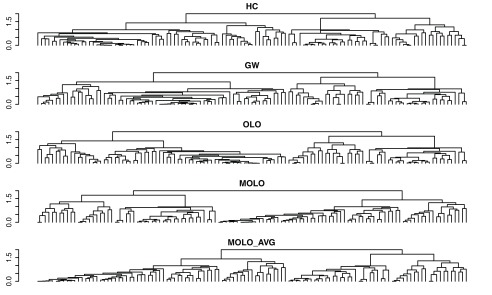
Comparison of dendrogram structures resulting from different leaf ordering methods. The rows from the example data sets are shown.

Similarly to previous examples, the application of the MOLO method results in a global right triangular shape for each subtree, encoding the monotonicity of the hierarchical clustering process ( Figure 7). However, upon a closer examination, we find that the first subtree of the rows does not form a right triangular shape. This first cluster is a very loose cluster having relatively long branches, except for the very first two rows which have the shortest distance. The characteristic of a loose cluster is also reflected in the heat map visualization, where there are no strong patterns of clustering, except for the first two rows. In order to prioritize tighter clusters with a smaller average distance, we implemented a variation of the modular leaf ordering method based on the average distance of the preceding merges (MOLO_AVG). The effects of leaf ordering methods on dendrogram structures for the rows are compared in Figure 8. With the MOLO_AVG method, the tight clusters with lower average distances are placed leftmost.

**Figure 9.**
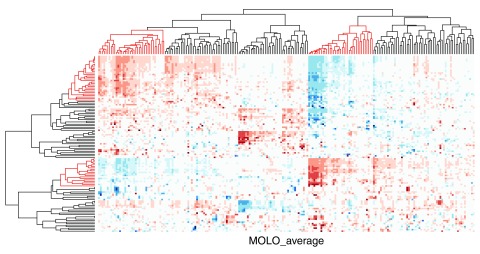
Cluster heat map of the data matrix after applying the MOLO method based on the average distance. The rows and columns with an inverse relationship are highlighted in the dendrograms.

The cluster heat map generated with the MOLO_AVG method is shown in Figure 9. The choice of either the smallest or average distance does not influence the structure within subtrees, however the order of the subtree structures changes. Although the difference may be subtle, we find that the modularity of clusters becomes more distinctive with the MOLO_AVG method. The resulting visualization also provides new insights into relationships between clusters. For instance, the inverse relationship between sets of rows and columns becomes more apparent in Figure 9 than the original figure ( Figure 1).

One way to evaluate the efficiency of a graphical representation is to compare the proportion of ink used to represent the data, a concept known as the data-ink ratio ^20^. Since each dendrogram shares the same underlying hierarchical clustering output, the total length of lines required to draw a dendrogram can be directly compared to evaluate the conciseness of dendrogram representations. We calculated the total length of lines used to draw dendrograms in Figure 9, the results of which are shown in Table 1.

**Table 1.** Comparison of the total line lengths required to draw the dendrogram structures shown in the Figure 8.

Method	HC	GW	OLO	MOLO	MOLO_AVG
Total length	559.79	598.93	551.98	492.88	437.48
Ratio to HC	1	1.07	0.99	0.88	0.78

The MOLO_AVG method results in the highest reduction in the data-ink ratio, while the GW method results in an increase in the data-ink ratio. Since the total number of vertical lines in each dendrogram is the same, the difference in the total length is due to the horizontal lines. A factor contributing to the reduction of horizontal lines is the heuristic of placing the singleton cluster on the right side of the branch. This heuristic avoids the placement of a singleton cluster on the left side, spreading over the nested tree structure.

**Figure 10.**
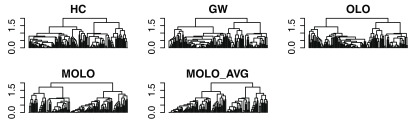
Comparison of dendrogram structures resulting from different leaf ordering methods in a limited display space. The rows from the example data sets are shown.

As the data size increases, the number of rows or columns in the data matrix increases while the display space for the figure may be limited. As a result, a dendrogram representation may become denser with more leaves, making the details of hierarchical structure harder to read. Figure 10 shows the same dendrograms as in Figure 8, but in a more limited display space. Because the MOLO methods results in a global pattern of right triangular shapes, it supports the viewer to identify tight and loose clusters even when the vertical lines of branches are so dense that they are in contact with adjacent branches. Similarly, because of this right triangular shape, each subtree structure is still distinguishable. Therefore, the MOLO methods aid the readability of dendrogram structures, even when the display size is limited.

In summary, this case study demonstrates how the MOLO methods support tasks in exploratory data analysis and improve readability of the dendrogram representations by reducing visual clutter. The dendrogram structure after the MOLO methods results in right triangular shapes for each subtree structure, and the order of leaves in each subtree reflects the order in which clusters are merged. In common with the case study of the Iris data set, the MOLO methods aid cluster identification in cluster heat maps.

## Discussion

In this paper, we introduce two modular leaf ordering methods and demonstrate how leaf ordering of dendrograms can influence the interpretation of cluster heat map visualizations. While seriation-based leaf ordering methods focus on homogenizing the linear order of leaves, our heuristics focus on improving the graphical representation of dendrograms to reflect the intrinsic properties of the hierarchical clustering process, such as the monotonic increase of distances in successive merges. As a result, each subtree structure has a global right triangular shape. This modular property is also reflected in the linear order of leaves, thus influencing the visual impression of clusters in heat map visualizations.

Although the leaf ordering methods affect the dendrogram representation and the linear order of leaves, it does not change the underlying hierarchical structure. In other words, the quality of the clustering results ultimately depends on the quality of the input data and the choice of appropriate distance metric and linkage algorithm. Given no prior knowledge of underlying patterns in data sets, it is recommended to try different normalization techniques in preprocessing and different distance measures and linkage algorithms to allow different aspects of the data to be explored ^14^.

## Conclusions

Through case studies, we demonstrate the effects of our leaf ordering methods on the interpretation of the clustering result, as well as the reduction in visual clutter as measured by the data-ink ratio. With cluster heat map techniques being very popular in life sciences, we advocate our methods to be considered both for exploratory data analysis and for publication of figures.

## Software availability

### Software access

http://cran.r-project.org/web/packages/dendsort/index.html

### Latest source code

https://bitbucket.org/biovizleuven/dendsort/

### Source code as at the time of publication

https://bitbucket.org/F1000Research/dendsortarchive

### Archived source code as at the time of publication

http://dx.doi.org/10.5281/zenodo.10980 ^21^

### Software license

GPL-2 | GPL-3

## References

[1] Wilkinson L Friendly M: The History of the Cluster Heat Map. *Am Stat.* 2009;63(2):179–184 10.1198/tas.2009.0033

[2] Gehlenborg N O’Donoghue SI Baliga NS: Visualization of omics data for systems biology. *Nat Methods.* 2010;7(3 Suppl):S56–68 10.1038/nmeth.1436 20195258

[3] de Souto MC Costa IG de Araujo DS: Clustering cancer gene expression data: a comparative study. *BMC Bioinformatics.* 2008;9:497 10.1186/1471-2105-9-497 19038021 PMC2632677

[4] Hastie T Tibshirani R Friedman J: The Elements of Statistical Learning. *Springer Series Statistics.* 2009 10.1007/978-0-387-84858-7

[5] Tan P Kumar V Steinbach M: Introduction to data mining. Boston: Pearson Addison Wesley, 1st ed edition.2005 Reference Source

[6] Bar-Joseph Z Gifford DK Jaakkola TS: Fast optimal leaf ordering for hierarchical clustering. *Bioinformatics.* 2001;17(Suppl 1):S22–9 10.1093/bioinformatics/17.suppl_1.S22 11472989

[7] Gehlenborg N Wong B: Points of view: Heat maps. *Nat Methods.* 2012;9(3):213 10.1038/nmeth.1902 27974286

[8] Morris SA Asnake B Yen GG: Dendrogram seriation using simulated annealing. *Information Visualization.* 2003;2(2):95–104 10.1057/palgrave.ivs.9500042

[9] James G Witten D Hastie T: An Introduction to Statistical Learning, of Springer Texts in Statistics. Springer New York, New York, NY.2013; **103** 10.1007/978-1-4614-7138-7

[10] Gruvaeus G Wainer H: Two Additions to Hierarchical Cluster Analysis. *J Math Stat Psychol.* 1972;25(2):200–206 10.1111/j.2044-8317.1972.tb00491.x

[11] Chae M Chen JJ: Reordering hierarchical tree based on bilateral symmetric distance. *PLoS One.* 2011;6(8):e22546 10.1371/journal.pone.0022546 21829631 PMC3150382

[12] Hahsler M Hornik K Buchta C: Getting Things in Order: An Introduction to the R Package seriation.2001 Reference Source

[13] Eisen MB Spellman PT Brown PO: Cluster analysis and display of genome-wide expression patterns. *Proc Natl Acad Sci U S A.* 1998;95(25):14863–14868 10.1073/pnas.95.25.14863 9843981 PMC24541

[14] Quackenbush J: Computational analysis of microarray data. *Nat Rev Genet.* 2001;2(6):418–27 10.1038/35076576 11389458

[15] Buchta C Hornik K Hahsler M: Getting things in order: an introduction to the R package seriation. *J Stat Softw.* 2008;25(3). Reference Source

[16] R Core Team and contributors worldwide. R: Edgar Anderson’s Iris Data.

[17] Ware C: Information Visualization: Perception for Design. Morgan Kaufmann Publishers Inc., San Francisco.2004 Reference Source

[18] Ware C: Color sequences for univariate maps: theory, experiments and principles. *IEEE Comput Graph Appl.* 1988;8(5):41–49 10.1109/38.7760

[19] The Cancer Genome Atlas Research Network. Comprehensive Molecular Characterization of Gastric Adenocarcinoma. *Nature.* 2014 10.1038/nature13480 PMC4170219 25079317

[20] Tufte ER: The Visual Display of Quantitative Information. Graphics Press, Cheshire, CT, USA.1986 Reference Source

[21] Sakai R Winand R Verbeiren T: R package dendsort for modular leaf ordering methods. *Zenodo.* 2014 Data Source 10.12688/f1000research.4784.1 PMC4162509 25232468

