# Supplementary material for: dendsort: modular leaf ordering methods for dendrogram representations in R
Source: F1000Res. 2014 Jul 30;3:177. [Version 1] doi: 10.12688/f1000research.4784.1 (PMC4162509; doi:10.12688/f1000research.4784.1)
Supplement: Peer review history

# Peer review: Referee response for version 1

**DOI:** 10.5256/f1000research.5108.r5926

**Published:** 2014-09-09

**Authors:** Jan Oosting

**Affiliations:** 1 Department of Pathology, Leiden University Medical Center, Leiden, Netherlands

The manuscript describes a method to improve the interpretation of dendrograms from clustering algorithms.

It is well written and the authors go into sufficient detail to describe the issues with the available ways to display dendrograms. They have applied their method to a number of case studies, and here they show that their method improves the distinction between clusters in the data.

The method is made available in the form of an R package (dendsort). Creating re-ordered dendrograms is quite easy with this package, but I found it more cumbersome to apply the method to heatmaps. The standard heatmap() function has a 'reorderfun' parameter, but it expects other parameters than the dendsort() function. The documentation of the package should be improved to make this easier.

I have read this submission. I believe that I have an appropriate level of expertise to confirm that it is of an acceptable scientific standard.

# Peer review: Referee response for version 1

**DOI:** 10.5256/f1000research.5108.r5627

**Published:** 2014-08-22

**Authors:** Eamonn Maguire, Rodrigo Santamaria

**Affiliations:** 1 Department of Computer Science, University of Oxford, Oxford, UK; 2 Department of Computer Science, University of Salamanca, Salamanca, Spain

This is an excellent piece of work which is immediately available to the bioinformatics community via the implementation in R and deployment to the CRAN repository.

Within the visualization community, we know that heat map representations work much better when clustered appropriately since correlations with colour are more difficult to detect when colour grouping is not present, see Haroz and Whitney (2012). This technique presented by the authors goes some way to making heat map representations more effective.

Their evaluation is complete and shows clear advantages over existing methods.

We have read this submission. We believe that we have an appropriate level of expertise to confirm that it is of an acceptable scientific standard.
